# First- and Second-Trimester Reference Intervals for Thyroid Hormones during Pregnancy in “Rhea” Mother-Child Cohort, Crete, Greece

**DOI:** 10.4061/2011/490783

**Published:** 2011-12-04

**Authors:** Polyxeni Karakosta, Leda Chatzi, Emmanouil Bagkeris, Vasiliki Daraki, Dimitris Alegakis, Elias Castanas, Manolis Kogevinas, Marilena Kampa

**Affiliations:** ^1^Department of Social Medicine, Faculty of Medicine, University of Crete, P.O. Box 2208, 71003 Heraklion, Greece; ^2^Department of Experimental Endocrinology, Faculty of Medicine, University of Crete, P.O. Box 2208, 71003 Heraklion, Greece; ^3^Department of Endocrinology, Faculty of Medicine, University of Crete, 71003 Heraklion, Greece; ^4^Centre for Research in Environmental Epidemiology (CREAL), Doctor Aiguader, 88, 08003 Barcelona, Spain; ^5^National School of Public Health, Alexandras Avenue 196, 115 21 Athens, Greece

## Abstract

Estimation and interpretation of thyroid function tests in pregnant women is of utmost importance for maternal, fetal and neonatal health. Our objective was to calculate laboratory- and geography-specific reference intervals for thyroid hormones during pregnancy in an iodine-sufficient area of the Mediterranean, Crete, Greece. This project was performed in the context of “Rhea” mother-child cohort. Fulfillment of extensive questionnaires and estimation of free triiodothyronine (fT3), free thyroxine (fT4), thyroid-stimulating hormone (TSH), and antithyroid antibodies were performed. The reference population was defined using inclusion criteria regarding thyroidal, obstetric, and general medical status of women. Reference interval for TSH was 0.05–2.53 **μ**IU/mL for the first and 0.18–2.73 **μ**IU/mL for the second trimester. 6,8% and 5,9% of women in the first and second trimester, respectively, had TSH higher than the upper reference limit. These trimester-specific population-based reference ranges are essential in everyday clinical practice for the correct interpretation of thyroid hormone values and accurate classification of thyroid disorders.

## 1. Introduction

Pregnancy is a period of significant hormonal changes and metabolic demands which result in complex effects on thyroid function [1–3]. More specifically, alterations in iodine metabolism [1], production of *β*-chorionic gonadotropin (*β*-hCG), and increases in both thyroid hormone-binding proteins and thyroid hormones *per se *[4, 5] are some of the physiologic changes that occur during normal pregnancy. At the same time, thyroid hormones play a critical role in neonatal and child neurodevelopment [6], and maternal thyroid disorders can lead to obstetric complications and irreversible effects on the fetus [7]. These findings point out the need for all pregnant women to be screened for thyroid disorders with a valid biomarker with distinct reference ranges.

In the past years, a number of studies from different regions have developed reference ranges for thyroid hormones during pregnancy women [8–31]; however these results should not be extrapolated due to differences in ethnicity, iodine intake, and immunometric assay applied in each study. Moreover, the methodology used for the determination of the reference population (choice of reference population, sample size, assessment of outliers) differs across studies resulting in a variation of absolute reference limits.

The aim of this study was to develop laboratory- and geography-specific reference intervals for thyroid hormones (thyroid-stimulating hormone (TSH), and free triiodothyronine (free T3), free thyroxine (free T4)) during pregnancy in an iodine-sufficient area of the Mediterranean, Crete, Greece.

## 2. Materials and Methods

### 2.1. Study Population

This project utilized data from the Rhea mother-child cohort, in the island of Crete, Greece. The mother-child “Rhea” study is a prospective cohort study examining a population sample of pregnant women and their children in a prefecture of southern Greece. Pregnant women, who became pregnant within one year, starting February 2007, participated in the study. The first contact was done at the time of the first major ultrasound, and women were divided in trimesters of pregnancy, according to gestational age which was defined by last menstrual period and ultrasound (first: <13 weeks, second: 13–27 weeks, and third trimester: >28 weeks). Participants were interviewed, and blood samples were collected and stored in −80°C. Extensive questionnaires were completed, and standardized information from ultrasounds was collected together with data from clinical records during pregnancy and birth. The study was approved by the corresponding ethical committees, and all participants provided written informed consent.

**Figure 1:**
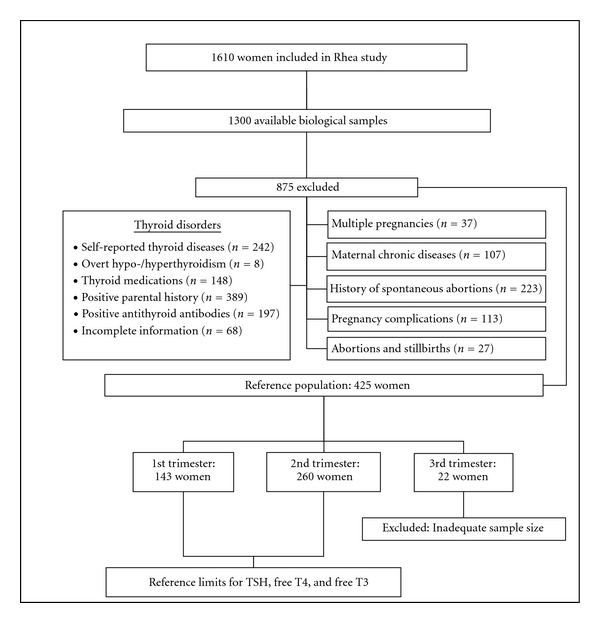
Flow diagram of the study process for the determination of the reference population.

From the entire population of the Rhea cohort (*n* = 1610), all available serum samples were analyzed for thyroid hormone measurements (*n* = 1300). According to the recommendations of the National Academy of Clinical Biochemistry (NACB) [32], we subsequently excluded women with a self-reported thyroidal dysfunction (goiter, cancer, hyper-, and hypo-thyroidism), a laboratory diagnosis of overt hypo- or hyperthyroidism (i.e., abnormal values of TSH and FT4 using the reference ranges of the assay used), evidence for autoimmune thyroid disease (elevated anti-TPO and anti-Tg), past or present use of thyroid medications, parental history of any thyroid illness, and women with incomplete information regarding thyroid function. In addition, women with multiple or complicated pregnancies (hyperemesis, gestational diabetes or hypertension, perinatal infections, and stillbirths), clinical diagnosis of a chronic or autoimmune disease (diabetes, hypertension, asthma, inflammatory bowel disease, tumors, and others), and a past history of spontaneous abortions were also removed from the reference population (Figure 1).

### 2.2. Laboratory Analysis

For each sample, TSH, free thyroxine (free T4), free triiodothyronine (free T3), and antithyroid antibodies (antithyroperoxidase [anti-TPO] and antithyroglobulin [anti-Tg]) were measured by IMMULITE 2000 immunoassay system (Siemens Healthcare Diagnostics, ILL 60015-0778, USA). For TSH, inter- and intra-assay variability were <5.3% and <6.4%, respectively, for levels of 0.32–39 mIU/mL. Accordingly, for free T4 these values were <7.8% and <7.1% for the level of 0.51–4.82 ng/dL (6.56–62.03 pmol/L), for free T3 < 9.1% & <10% for the level of 2.5–13 pg/mL (3.84–19.96 pmol/L), for anti-Tg < 4.9% and <5.8%, and for anti-TPO < 7.4% and 7.2%. The proposed reference limits of the manufacturer for normal euthyroid adults were: free T3: 1.8–4.2 pg/mL (2.76–6.45 pmol/L), free T4: 0.89–1.76 ng/dL (11.5–22.7 pmol/L), and TSH: 0.4–4 *μ*IU/mL. Anti-TPO and anti-Tg were considered elevated if levels were ≥35 IU/mL and >40 IU/mL, respectively.

### 2.3. Statistical Analysis

All data were analyzed by SPSS 17 for windows. The nature of the underlying distribution of free T3, free T4, and TSH for the reference population was examined by inspecting normality tests, histograms and P-plots. In case of a significant variation from normal distribution, a logarithm transformation (log and ln) was applied to achieve normality. Outliers were identified using box plots. For the identified outliers Dixon's *Q* test was applied to the least extreme; if the test rejects the least extreme outlier, then the more extreme outliers are also rejected. Continuously, when the data followed a Gaussian distribution or were transformed to a normal distribution, reference intervals were computed as follows: mean ± 1.96 × standard deviation. If normality was not achieved, even after transformation or after the outlier deletion, a nonparametric method was applied to estimate the reference intervals, by computing the rank numbers of 2.5th and the 97.5th percentiles to estimate the lower and the higher limits of the reference interval, respectively.

Thyroid hormones were expressed as mean, median, standard deviation, 2.5th and 97.5th percentile for the 1st and 2nd trimester. The Mann-Whitney *U* test was used to compare differences for the 2 trimesters for a level of significance of *P* < 0.05.

## 3. Results

### 3.1. Total Study Population

Starting from a total cohort population of 1610 pregnant women, 1300 samples were available for thyroid function and antibody analyses. Of them, 35.2% were in the first (<13 weeks), 61% in the second (13–27 weeks), and 3.7% in the third trimester (>28 weeks) of pregnancy (Table 1). The age of the mothers varied from 15 to 45 years, and the majority of mothers were of Greek origin (85.3%). History of spontaneous miscarriages was present in 223 women (17.2%). Considering thyroid function, 389 (29.9%) of mothers had a positive family history of thyroidal disease, while 165 (12.7%) and 87 (6.7%) women had elevated levels of anti-TPO and anti-TG antibodies, respectively.

### 3.2. Reference Population

After implementation of the aforementioned exclusion criteria, a total of 875 women were excluded from the study (Figure 1, Table 1), resulting to a final population of 425 women (1st trimester: 143, 2nd trimester: 260, 3rd trimester: 22). Women in the third trimester (*n* = 22) were excluded from the analysis, since the sample size was not adequate for the estimation of reference intervals to a reasonable degree of precision. The final population (403 women) was used to determine the reference limits and the 95% confidence intervals for TSH, free T4, and free T3 for the first and second trimester of pregnancy.

### 3.3. Reference Values for Thyroid Hormones

**Figure 2:**
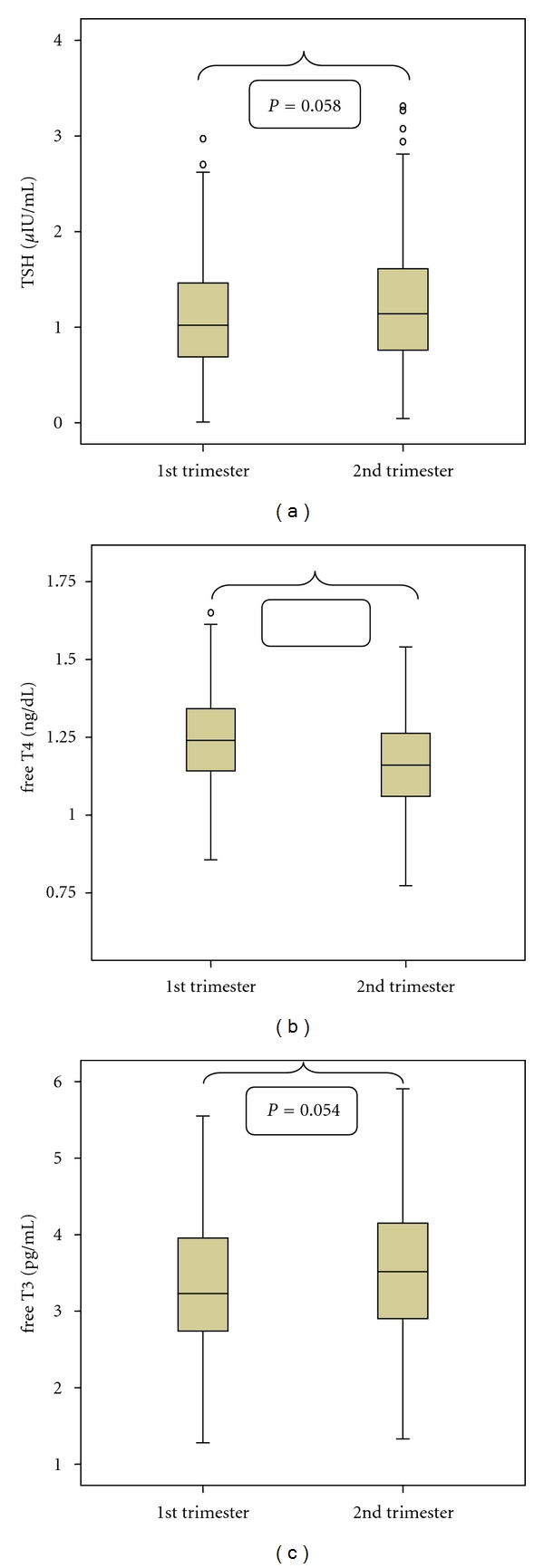
Box plot of TSH, free T3, and free T4 concentrations in the reference population during the 1st and 2nd trimester of gestation. TSH: thyroid-stimulating hormone; Free T3: free triiodothyronine; free T4: free thyroxine Differences between first and second trimester are shown with *P* values (*P* = 0.058 for TSH, *P* = 0.054 for free T3, *P* < 0.001 for free T4).

Figure 2 shows the box plots for TSH, free T3, and free T4 for first and second trimester after the outlier assessment. Additionally, mean values, standard deviations, medians, and the 2.5th and 97.5th percentiles for all thyroid hormones according to trimester are shown in Table 2.

According to our results (Tables 2 and 3), the reference intervals of serum TSH levels for the first trimester were 0.05–2.53 *μ*IU/mL, of free T3 1.54–5.22 pg/mL (2.37–8.02 pmol/L), and of free T4 0.95–1.53 ng/dL (12.23–19.69 pmol/L). For the second trimester, respective reference intervals were: 0.18–2.73 *μ*IU/mL for TSH, 1.78–5.29 pg/mL (2.73–8.13 pmol/L) for free T3, and 0.87–1.45 ng/dL (11.20–18.66 pmol/L) for free T4. Median TSH and free T3 values showed a slight increase in 2nd trimester, while median free T4 values fell as gestational age advanced.

Mann Whitney *U* test revealed significant differences between trimesters for free T4 (*P* value < 0.001), while respective *P* values for TSH and free T3 were 0.058 and 0.054. These findings justify the separation of groups into different trimesters (Table 2, Figure 2).

As shown in Table 4, if the reference limits of the manufacturer were applied to our entire cohort, misclassification of maternal thyroid clinical entities would occur. More specifically, 47 and 43 women with TSH concentrations that are normal for the first and second trimester, respectively would have been misclassified as having subclinical hyperthyroidism. Conversely, 25 and 29 women with a TSH above the first and second trimester-specific upper reference limit would not have been identified as subclinical hypothyroidism.

## 4. Discussion

During pregnancy several hormonal changes and metabolic demands occur, resulting in complex effects on thyroid function [3]. Alterations in the pituitary-thyroid axis include an increase in thyroid hormone-binding globulin along with increases in total T4, T3 as well as serum thyroglobulin (TG). Additionally, iodine clearance by the kidneys is enhanced during gestation, while the mild thyrotropic effects of rising *β*-hCG may exert negative feedback on TSH secretion [33] wrongly suggesting hyperthyroidism in normal pregnant women of the 1st trimester [1].

The incidence of overt and subclinical hypothyroidism in pregnant women has been estimated to be around 0.3–0.5 and 2-3%, respectively [34]. Recent studies have shown that untreated hypothyroidism during pregnancy increases the incidence of maternal anemia, preeclampsia, postpartum hemorrhage, placental abruption, and spontaneous abortion and may cause low birth weight, prematurity, congenital malformations, and impaired fetal brain development with decreased intelligence quotient (IQ) of children [35]. Conversely, hyperthyroidism has been described in about 0.2% of women during pregnancy [36] and may lead to preeclampsia, stillbirths, preterm delivery, intrauterine growth retardation, and low birth weight [7]. Withstanding the above, the serial changes in serum thyroid hormone levels imply the need to better define “pregnancy-specific” normative reference ranges for thyroid function tests for early diagnosis of hyper- and hypothyroidism during pregnancy.

Our study represents the first study performed in a Mediterranean area, the island of Crete, within an iodine-sufficient country, Greece [37]. It provides reference ranges for thyroid hormones during the first and second trimester of pregnancy. International guidelines recommend determining serum TSH as the first-line screening variable for thyroid dysfunction before conception [38] and during pregnancy [3, 39]. According to our results and in agreement with previous studies [40], the derived reference intervals for TSH were different (narrower and lower) from those proposed by the manufacturer. More specifically, our TSH reference intervals were 0.05–2.53 and 0.18–2.73 *μ*IU/mL for the first trimester and second trimester, respectively, compared to 0.4–4 *μ*IU/mL. Consequently, women with subclinical hypothyroidism would not have been identified, and normal women would have been misclassified as having subclinical hyperthyroidism if the manufacturer's TSH limits were used. Regarding free T4, and free T3, our intervals were only slightly different.

Many cross-sectional studies have reported trimester-specific reference ranges for free T3, free T4, and TSH among pregnant women [8–31]. However these reported reference ranges vary due to differences in ethnicity, iodine intake, sample size, assessment of reference population, and immunometric assay used among studies.  Table 5 summarizes 1st and 2nd trimester-specific reference intervals for thyroid hormones from 21 studies worldwide. Ethnic disparities along with variations in iodine nutrition characteristics result in geographic variability of hormonal values. In addition, different reagents used by laboratories recognize distinct circulating TSH isoforms with resulting fluctuations even for the same sample [41]. Therefore, there is a growing need for laboratory- and geography- specific reference intervals [42]. Moreover, the methodology used by the published studies to date varies in terms of inclusion criteria for the determination of reference population, sample size, and assessment of outliers. More specifically, most studies used nonparametric methods in order to provide reference intervals without reporting the underlying distribution [10, 12, 14, 19], did not mention whether outliers were detected and removed [10–20], and most importantly, in some cases, have not applied strict criteria to obtain a well-defined healthy population [13, 14, 16, 17]. According to the National Academy of Clinical Biochemistry (NACB) [32] and the National Health and Nutrition Examination Survey (NHANES), this well-defined healthy population should be based on specific exclusion criteria and represents the most important prerequisite for the determination of reference intervals [43, 44].

 Based on the above, we selected our reference population from a large pool of Rhea mother-child cohort in Crete, after implementation of stringent criteria. Initially, we excluded all mothers with any kind of thyroid abnormality since women positive for thyroid autoantibodies typically have higher TSH values and therefore affect and skew the upper reference limit [45, 46]. In addition, women with twin pregnancies or with hyperemesis gravidum were also removed from the reference population due to their potential for low TSH values (higher serum hCG) and interference with the lower limit of TSH reference range [22, 47, 48]. Based on the association between autoimmunity and thyroid dysfunction [49], we also excluded mothers with positive history of autoimmune diseases. The final strictly defined reference population of 403 women was considered adequate for the estimation of reference intervals fulfilling the sample size requirements of Clinical Laboratory Standards (NCCLS) [43]. Additional methodological strengths of our study include examination of distributions and respective application of parametric or nonparametric methods, assessment of outliers, and use of a statistical test to resolve whether separate reference intervals should be calculated for the first and second trimester. Results from this test confirmed the need for trimester-specific reference ranges in agreement to the existing literature and as indicated by normal physiology.

Our study is limited by lack of data concerning the third trimester. Pregnant women were partitioned into trimesters upon entering the study, and thus the resulting sample size (*n* = 22) was not adequate for the estimation of reference intervals to a reasonable degree of precision. However, during the second half of gestation TSH levels return to prepregnancy levels and remain stable [50]. In addition, there was only a small number of women (*n* = 12) in less than 8 weeks of gestation, when hCG has a minimal effect on thyroid. Contrary to The National Health and Nutrition Examination Survey in the USA [36], some studies propose that thyroid ultrasound should be used as an additional exclusion criterion to rule out thyroid pathology [51]. In our study, we did not include thyroid ultrasound for the detection of goiter or presence of hypoechogenicity and nodularity of thyroid, since these data were not collected. Finally, an important limitation is the assumption of iodine sufficiency in all women, as we did not evaluate iodine status by urine iodine estimation. However, median urinary iodine excretion (the best parameter to evaluate the adequacy of iodine nutrition in a population) during the last two decades in Greece has been estimated to be over 200 *μ*g/g Cr [52, 53], which is well within normal limits [54]. These findings indicate that, at present, Greece may be considered as an iodine-sufficient country.

## 5. Conclusion

Data from this study establish reference values for Greek Cretan pregnant women and point out the need for laboratory- and geography-specific reference ranges in an effort to detect overt and subclinical thyroid disorders and to evaluate the risk for both obstetric complications and impaired fetal development.

## Figures and Tables

**Table 1:** Demographic data for pregnant women in total and reference study population.

	Total study population^a^			Reference population	
	1st trimester	2nd trimester	3rd trimester	1st trimester	2nd trimester
Sample size (*n*)	458	794	48	143	260
Age (years)^b^	29.16 (5.12)	28.98 (5.19)	28.37 (4.61)	28.50 (5.09)	27.79 (4.95)
Greek origin *n* (%)	369 (80.6)	698 (87.9)	42 (87.5)	114 (79.7)	221 (85)

^a^Women with available biological samples.

^b^Values are expressed as mean (SD).

**Table 2:** Thyroid hormones according to gestational age, Rhea mother-child cohort, Crete, Greece.

	*N*	Distribution	2.5th percentiles	97.5th percentile	Median	Mean	SD	Reference range
1st trimester								
TSH^a^ *μ*IU/mL	141	Not normal	0.05	2.53	1.02	1.08	0.61	0.05–2.53
free T3^b^ pg/mL (pmol/L)	141	Normal^d^	1.84 (2.83)	5.39 (8.28)	3.21 (4.93)	3.38 (5.19)	0.94 (1.44)	1.54–5.22 (2.37–8.02)
free T4^c^ ng/dL (pmol/L)	139	Normal^e^	0.96 (12.36)	1.60 (20.59)	1.24 (15.96)	1.24 (15.96)	0.15 (1.93)	0.95–1.53 (12.23–19.69)

2nd trimester								
TSH^a^ *μ*IU/mL	257	Not normal	0.18	2.73	1.14	1.23	0.65	0.18–2.73
free T3^b^ pg/mL (pmol/L)	256	Normal	1.99 (3.06)	5.30 (8.14)	3.52 (5.41)	3.55 (5.45)	0.87 (1.34)	1.78–5.29 (2.73–8.13)
free T4^c^ ng/dL (pmol/L)	258	Normal	0.84 (10.81)	1.44 (18.53)	1.16 (14.93)	1.16 (14.93)	0.15 (1.93)	0.87–1.45 (11.20–18.66)

TSH: thyroid-stimulating hormone; free T3: free triiodothyronine; free T4: free thyroxine.

^a^Difference between first and second trimester: *P* = 0.058.

^b^Difference between first and second trimester: *P* = 0.054.

^c^Difference between first and second trimester: *P* < 0.001.

^d^After outlier deletion and log transformation.

^e^After outlier deletion.

**Table 3:** Comparison between reference ranges for thyroid hormones as calculated by our analysis and those proposed by the manufacturer.

Reference ranges	TSH (*μ*IU/mL)		free T4 (ng/dL)		free T3 (pg/mL)	
	1st trimester	2nd trimester	1st trimester	2nd trimester	1st trimester	2nd trimester
Rhea cohort	0.05–2.53	0.18–2.73	0.95–1.53	0.87–1.45	1.54–5.22	1.78–5.29
Manufacturer	0.4–4		0.89–1.76		1.8–4.2	

TSH: thyroid-stimulating hormone; free T3: free triiodothyronine; free T4: free thyroxine.

**Table 4:** Thyroid clinical entities in the general population of Rhea mother-child cohort based on: (i) the manufacturer's and (ii) the derived reference limits.

Trimester	Reference limits	Normal thyroid function *n* (%)^a^	Subclinical hypothyroidism *n* (%)^b^	Clinical hypothyroidism *n* (%)^c^	Subclinical hyperthyroidism *n* (%)^d^	Clinical hyperthyroidism *n* (%)^e^
1st	Manufacturer	386 (84.3)	6 (1.3)	0 (0)	48 (10.5)	7 (1.5)
	Rhea cohort	371 (81.0)	31 (6.8)	0 (0)	1 (0.2)	10 (2.2)
2nd	Manufacturer	680 (86.6)	18 (2.3)	1 (0.1)	61 (7.7)	1 (0.1)
	Rhea Cohort	653 (82.2)	47 (5.9)	0 (0)	18 (2.3)	9 (1,1)

^a^TSH between reference limits and free T4 between reference limits.

^b^TSH over the upper limit of reference limits and free T4 between reference limits.

^c^TSH over the upper limit of reference limits and free T4 under the lower limit of reference limits.

^d^TSH under the lower limit of reference limits and free T4 between reference limits.

^e^TSH under the lower limit of reference limits and free T4 over the upper limit of reference limits.

**Table 5:** Worldwide summary of studies reporting 1st and 2nd trimester-specific reference intervals for thyroid hormones during pregnancy.

Study	Country (subgroups)	Sample size	Exclusion criteria	Assay used/test (units)	Reference limits	
					1st trimester	2nd trimester
Price et al., 2001 [23]	UK (Caucasians/Asians)	120	(i) Serious gestational events (ii) Endocrinological medication	CL (ACS 180, Bayer) TSH (mIU/L) Free T4 (pmol/L)	Caucasians (*n* = 50): TSH: 0.7–1.1 Free T4: 12.0–12.8 Asians (*n* = 20): TSH: 0.6–1.3 Free T4: 11.8–13.4	Caucasians (*n* = 50): TSH: 1.2–1.5 Free T4: 11.2–11.8 Asians (*n* = 20): TSH: 1.0–1.8 Free T4: 10.9–12.1

Panesar et al., 2001 [9]	China	406	(i) Thyroid disease (ii) Serious gestational events (incl. hyperemesis, trophoblastic disease, preeclampsia)	CL (ACS 180, Chiron Diagn.) TSH (mIU/L) Free T4 (pmol/L) Free T3 (pmol/L)	—^a^	—^a^

Haddow et al., 2004 [13]	USA	1126	(i) Thyroid Ab(+)	CL (Immulite, Siemens) TSH (mIU/L)	(*n* = 1005) TSH: 0.08–2.73	(*n* = 1005) TSH: 0.39–2.70

Kurioka et al., 2005 [14]	Japan	522	(i) Multiple pregnancies	ECL (Elecsys, Roche) TSH mIU/mL Free T4 (ng/dL) Free T3 (pg/mL)	(*n* = 119) TSH: 0.04–3.39 Free T4: 1.16–1.95 Free T3: 2.68–4.59	(*n* = 132) TSH: 0.17–3.72 Free T4: 0.89–1.39 Free T3: 2.56–4.11

Dhatt et al., 2006 [24]	United Arab Emirates (United Arabs/Other Arabs/Asians)	1140	(i) Multiple pregnancies (ii) Thyroid Ab(+) (iii) Thyroid disease (iv) Thyroid medication (v) Serious gestational events (incl. hyperemesis)	CL (Architect, Abbott) TSH (mIU/L) Free T4 (pmol/L)	United Arabs (*n* = 97): TSH: 0.06–8.3 Free T4: 8.9–24.6 Other Arabs (*n* = 122): TSH: 0.04–9.3 Free T4: 10.5–22.3 Asians (*n* = 79): TSH: 0.12–7.4 Free T4: 11.3–2.19	United Arabs (*n* = 252): TSH: 0.17–5.9 Free T4: 8.4–19.3 Other Arabs (*n* = 283): TSH: 0.23–5.7 Free T4: 9.5–18.7 Asians (*n* = 174): TSH: 0.3–5.5 Free T4: 9.7–18.5

Stricker et al., 2007 [12]	Switzerland	2272	(i) Thyroid Ab(+) (ii) Thyroid medication (iii) Miscarriage (iv) Fetal genetic abnormality	CL (Architect, Abbott) TSH (mIU/L) Free T4 (pmol/L) Free T3 (pmol/L)	(*n* = 783) TSH: 0.0888–2.8293 Free T4: 10.53–18.28 Free T3: 3.52–6.22	(*n* = 528) TSH: 0.1998–2.7915 Free T4: 9.53–15.68 Free T3: 3.41–5.78

La'ulu and Roberts, 2007 [19]	USA (Asians/Blacks/ Hispanics/Whites)	3064	(i) Thyroid Ab(+)	CL (Architect, Abbott) TSH (mIU/L) Free T4 (pmol/L) Free T3 (pmol/L)	—	(*n* = 2683)^b^ TSH: 0.15–3.11 Free T4: 9.3–15.2 Free T3: 3.83–5.96

Cotzias et al., 2008 [15]	UK	335	(i) Multiple pregnancies (ii) Thyroid/endocrine disease (iii) Serious gestational events (incl. preeclampsia, hyperemesis) (iv) Medication (v) Age < 15 & >45	CL (Advia Centaur, Siemens) TSH (mIU/L), Free T4 (pmol/L) Free T3 (pmol/L)	—^a^	—^a^

Lambert-Messerlian et al., 2008 [16]	USA	9562	(i) Multiple pregnancies (ii) Thyroid disease	CL (Immulite, Siemens) TSH (mIU/L)	(*n* = 9562) TSH: 0.13–4.15	(*n* = 9562) TSH: 0.36–3.77

Gong and Hoffman, 2008 [17]	Canada	340	(i) Thyroid Ab(+) (ii) TSH < 0.1 mIU/L (iii) TSH > 2.5 mIU/L (1st trimester) (iv) TSH > 3.0 mIU/L (2nd trimester) (v) TSH > 3.5 mIU/L (3rd trimester)	ECL (Modular E170, Roche) Free T4 (pmol/L)	(*n* = 224) Free T4: 11–19	(*n* = 240) Free T4: 9.7–17.5

Pearce et al., 2008 [25]	USA	668	(i) Multiple pregnancies (ii) Thyroid Ab(+) (iii) Thyroid disease (iv) Thyroid medication (v) TSH >5.5 mIU/L in Ab(−) women (vi) Miscarriages/fetal death	CL (Advia Centaur, Bayer) TSH (mIU/L)	(*n* = 585) TSH: 0.04–3.6	—

Marwaha et al., 2008 [11]	India	541	(i) Thyroid Ab(+) (ii) Thyroid disease (iii) Thyroid medication (iv) Family history of thyroid disease (v) Thyroid hypoechogenicity/nodularity (US) (vi) History of abortions and hyperemesis	ECL (Elecsys, Roche) TSH (mIU/mL) Free (T4 pmol/L) Free (T3 pmol/L)	(*n* = 107) TSH: 0.6–5.0 Free T4: 12–19.45 Free T3: 1.92–5.86	(*n* = 137) TSH: 0.435–5.78 Free T4: 9.48–19.58 Free T3: 3.2–5.7

Gilbert et al., 2008 [26]	Australia	2159	(i) Thyroid Ab(+) (ii) Thyroid disease	CL (Architect, Abbott) TSH (mIU/L) Free T4 (pmol/L) Free T3 (pmol/L)	(*n* = 1817) TSH: 0.02–2.15 Free T4: 10.4–17.8 Free T3: 3.3–5.7	—

Springer et al., 2009 [18]	Czech republic	5520	(i) Thyroid Ab(+) (ii) Thyroid diseases (iii) *β*-HCG > 56 mg/L	CL (Advia Centaur, Siemens) TSH (mIU/L)	(*n* = 4337) TSH: 0.06–3.67	—

Bocos-Terraz et al., 2009 [10]	Spain	1198	(i) Thyroid Ab(+) (ii) Thyroid disease	CL (Architect, Abbott) TSH (*μ*IU/mL) Free T4 (ng/dL) Free T3 (pg/mL)	—^a^	—

Ashoor et al., 2010 [27]	UK (Blacks/Whites)	4318	(i) Multiple pregnancies (ii) Thyroid Ab(+) (iii) Thyroid disease (iv) Serious gestational events (incl. preeclampsia, miscarriage/fetal death, delivery <34 weeks) (v) Major fetal abnormalities (vi) Birth weight < 5th percentile	CL (Advia Centaur, Siemens) TSH (mIU/L) Free T4 (pmol/L) Free T3 (pmol/L)	—^a^	—

Garía de Guadiana Romualdo et al., 2010 [29]	Spain	441	(i) Thyroid Ab(+) (ii) Thyroid disease incl. goiter (iii) Thyroid medication	ECL (Cobas, Roche) TSH (mUI/L) Free T4 (ng/dL)	(*n* = 400) TSH: 0.130–3.710 Free T4: 0.89–1.50	—

Yu et al., 2010 [30]	China	538	(i) Multiple pregnancies (ii) Thyroid disease incl. goiter (iii) Thyroid Ab(+) (iv) History of autoimmune diseases (v) Serious gestational events (incl. hyperemesis, hypertension, gestational diabetes, premature delivery) (vi) Medical history affecting thyroid function (vii) Abnormal urinary iodine	ECL (Cobas, Roche) TSH (mUI/L) Free T4 (pmol/L)	(*n* = 301) TSH: 0.02–3.65 Free T4: 11.85– 21.51	(*n* = 301) TSH: 0.36– 3.46 Free T4: 9.45–16.26

Männistö et al., 2011 [31]	Finland	5805	(i) Multiple pregnancies (ii) Thyroid Ab(+) (iii) Thyroid disease (iv) Thyroid medication	CL (Architect, Abbott) TSH (mIU/L) Free T4 (pmol/L) Free T3 (pmol/L)	**—** ^ a^	—^a^

Yan et al., 2011 [8]	China	505	(i) Multiple pregnancies (ii) Thyroid Ab(+) (iii) Thyroid disease incl. goitre (iv) Family history of thyroid disease (v) TSH > 5.0 mIU/l (vi) Serious gestational events (vii) Endocrinological medication	CL (Advia Centaur, Bayer) TSH (mIU/L) Free T4 (pmol/L) Free T3 (pmol/L)	(*n* = 168) TSH: 0.03–4.51 Free T4: 11.8–21 Free T3: 3.57– 5.61	(*n* = 168) TSH: 0.05–4.50 Free T4: 10.6–17.6 Free T3: 3.55– 5.25

Santiago et al., 2011 [28]	Spain	429	(i) Thyroid Ab(+) (ii) TSH > 5 *μ*IU/mL (iii) Major health problems	CL (Beckman access) TSH (*μ*IU/mL) Free T4 (ng/dL) Free T3 (pg/mL)	(*n* = 279) TSH: 0.23– 4.18 Free T4: 0.60–1.06 Free T3: 2.33–3.84	(*n* = 210) TSH: 0.36–3.89 Free T4: 0.43–0.85 Free T3: 2.04–3.51

^a^reference intervals other than trimester-specific (data not shown);

^b^combined reference interval for all ethnicities; reference intervals also provided for ethnicity subgroups (data not shown).

CL: Chemiluminescence assay; ECL: Electrochemiluminescence assay; TSH; thyroid-stimulating hormone; Free T4; serum-free thyroxine; Free T3; serum-free triiodothyronine;

to convert to SI units, for

FT4: pmol/L = ng/dL *x*  12.87

FT3: pmol/L = pg/mL *x*  1.536.
